# Informing the development of a decision aid: Expectations and wishes from service users and psychiatrists towards a decision aid for antipsychotics in the inpatient setting

**DOI:** 10.1111/hex.13749

**Published:** 2023-03-14

**Authors:** Katharina Müller, Florian Schuster, Silvia Krumm, Stefan Leucht, Spyridon Siafis, Stephan Heres, Peter Brieger, Johannes Hamann

**Affiliations:** ^1^ Department of Psychiatry and Psychotherapy, School of Medicine Technical University of Munich Munich Germany; ^2^ kbo‐Isar‐Amper‐Klinikum München Munich Germany; ^3^ Schön Klinik Bad Aibling Harthausen Bad Aibling Germany; ^4^ Department of Psychiatry II, Bezirkskrankenhaus Günzburg Ulm University Günzburg Germany; ^5^ Bezirkskrankenhaus Mainkofen Deggendorf Deutschland

**Keywords:** antipsychotic medication, decision aid, SDM, EBM

## Abstract

**Objectives:**

Decision aids (DAs) are promising tools to foster evidence‐based shared decision‐making between practitioners and service users. Nevertheless, it is still obscure how an evidence‐based DA for people with severe mental illness, especially psychosis, should look in an inpatient treatment setting to be useful and feasible. Therefore, we conducted focus groups with psychiatrists and service users to collect and assess their expectations and wishes regarding an evidence‐based DA. From these findings, we derived immediate recommendations for the future development of DAs.

**Methods:**

We held two group interviews with service users (*n* = 8) and three group interviews with psychiatrists (*n* = 10). We used an open, large‐scale topic guide. First, we presented data from a current meta‐analysis on antipsychotics to the interviewees and, in a second step, asked for their expectations and wishes towards a DA that integrates these data.

**Results:**

Our thematic analysis revealed six key themes addressed by the respondents: (1) general considerations on the importance and usefulness of such a DA, (2) critical comments on psychiatry and psychopharmacotherapy, (3) communicative prerequisites for the use of a DA, (4) form and content of the DA, (5) data input, data processing and output as well as (6) application of the DA and possible obstacles.

**Conclusions:**

Participants identified several important features for the development of DAs for selecting antipsychotics in inpatient psychiatric treatment. The digital format was met with the greatest approval. Especially the adaptability to different needs, users and psychopathologies and the possibility to outsource information dissemination via app seemed to be a decisive convincing argument. Further research is required to test specific features of DAs to be developed in clinical settings.

## Patient or Public Contribution

The entire project is supported by an advisory board, including also experienced users. The members are regularly updated about the process and give important feedback.

## 1 INTRODUCTION

Shared decision‐making (SDM) is a model for medical encounters in which patients and providers make decisions together as equals while sharing information, seeking consensus and ultimately agreeing on the treatment decision to be made.^1^, ^2^ In an initial review of SDM in psychiatry, Hamann et al.^3^ found a high desire for participation on the part of patients but several open questions regarding the feasibility of SDM in this setting. As SDM is already successfully practised in other medical fields and its positive impact on health‐related outcomes has already been demonstrated, they recommended the development of decision aids (DAs) as well as medical training in risk communication to overcome disease‐immanent and systemic barriers.^3^ Zisman‐Ilani et al.^4^ pointed out the tendency of research on SDM in mental health to focus on DAs to promote information sharing between patients and clinicians. At the same time, they also drew attention to approaches that focused more on the joint discussion of values and goals to strengthen the doctor–patient relationship. Strengthening the rapport was assessed as an important approach for implementing SDM in mental health, in line with findings from research on successful therapeutic processes. The authors suggested not to directly apply SDM outcomes from other medical fields. Rather, participation in SDM itself could be considered an important factor in mental health recovery as it promotes empowerment, whereas a reduction in decisional conflict or an increase in knowledge as a potential outcome is more difficult to achieve in this field given the many barriers.^5^

Successful implementation of SDM in mental health can be hampered by several barriers. Hamann and Heres^6^ described professionals' lack of time or staff's tendency to consider patients with acute psychotic episodes as unsuitable for SDM because of associated cognitive or emotional deficits. Barriers on the patients' side were described including passivity and loss of motivation.^6^ However, there is no evidence to date, that cognitive impairments prevent participation in SDM.^7^, ^8^ Zisman‐Ilani et al.^9^ point out the role of stigmatisation on the physician side towards patients with severe mental illness (SMI) as a likely barrier for SDM. Further barriers include distorted information given by physicians, for example, regarding age‐ or personality‐related aspects.^10^, ^11^ In addition, it is difficult for psychiatrists to keep track of the multitude of medications available, which also means that both sides are not always aware of the most current evidence on available treatment options.^12^

Against the background that antipsychotics often show similar efficacy but different side effect profiles, the intake of antipsychotics is a preference‐based decision that lends itself very well to the use of SDM.^13^ Although nonadherence rates are high with estimations between 40% and 60%,^14^ antipsychotics remain the treatment of choice for schizophrenia according to various treatment guidelines.^15^ DAs are promising tools for addressing the above‐mentioned problems. Nevertheless, only a few have been developed for the use of antipsychotics. In their scoping review, Müller et al.^16^ compared existing tools with the established quality criteria for patient‐based DAs with mixed results for the improvement of patient outcomes related to the use of a DA. In summary, the authors consider the current quality criteria for DAs for patients with schizophrenia as inadequate due to the complexities of factors influencing the decision for or against antipsychotics. In any case, they only found one DA that was clearly oriented on the International Patient Decision Aids Standards criteria, the ‘APM‐DA’ or ‘the Antipsychotic Medication Decision Aid’.^17^, ^18^

Testing of DAs for antipsychotics in inpatient settings is still rare, and existing tools are aimed at either service users or clinicians rather than involving both stakeholders in the process to address barriers on both sides.^19^, ^20^ To develop and test a new DA for antipsychotics in inpatient settings, a qualitative study was conducted to explore the expectations and desires for the DA to be developed amongst the two main stakeholders: People treated for psychosis and psychiatrists.

## 2 METHODS

### 2.1 Study design and setting

To investigate the wishes and expectations of psychiatrists and service users towards an evidence‐based DA, a qualitative design was chosen to explore the perspectives in the psychiatric inpatient setting of two hospitals in Munich, Germany. We chose to interview both parties involved separately, to allow the most open expression of opinions. The sample originally included 10 service users and 10 psychiatrists to ensure sufficient discussion and diversity of opinions. Due to the COVID‐19 pandemic restrictions, a maximum of five individuals were allowed to participate, resulting in a total of four interviews to achieve the sample size. One focus group was also split in two (centre 2), as several physicians were unable to attend at short notice during an agreed appointment. Therefore, five group interviews were held in the end. For more information on the dates and the exact number of participants in each focus group, see Table 1.

**Table 1:** Overview and assignment of all patient IDs to the respective focus groups.

Focus group					Focus group					
Psychiatrists	Date	Participant ID	Sex	Age	Service users	Date		Participant ID	Sex	Age
Group 1 (centre1)	12.10.20	G1Psychr01	M	33	Group 1 (centre 1)		21.10.20	G1SU01	M	32
		G1Psychr02	M	48				G1SU02	M	35
		G1Psychr03	M	33				G1SU03	F	28
		G1Psychr04	F	28				G1SU04	F	63
		G1Psychr05	F	31						
Group 2 (centre 2)	28.10.20	G2Psychr01	F	31	Group 2 (centre 2)		03.11.20	G2SU01	M	26
		G2Psychr02	F	36				G2SU02	F	56
Group 3 (centre 2)	17.11.20	G3Psychr01	M	37				G2SU03	M	31
		G3Psychr02	F	30				G2SU04	M	43
		G3Psychr03	M	35						

Abbreviations: M, male; W, women.

The first two focus groups were held in the same clinic where the research group is located (centre 1). For this reason, individual service users knew the interviewers, but no therapeutic relationship existed between the interviewers and study participants. For training purposes, the entire team of five researchers participated in the first group interview with psychiatrists in centre 1, consisting of the interviewer F. S., the assistant K. M., two chief physicians/project leaders and one other research assistant. In this case, the two project leaders present were also the supervisors of four of the five participating psychiatrists in their residency training. Afterwards, the research team reflected on the process and structure to inform the subsequent group interviews. On average, the group interviews lasted about 40 min. The study was approved by the local review board of the Technical University of Munich. For the reporting of the manuscript, we rely on the consolidated criteria for reporting qualitative health research checklists.^21^

### 2.2 Participants and recruitment

The inclusion criteria were ‘experience in the treatment of psychoses’ (psychiatrists) and ‘current inpatient treatment’ as well as a diagnosis of a schizophrenia spectrum disorder (International Classification of Diseases‐10 F20–F29; service users). A convenience sampling strategy was implemented by inviting all psychiatrists eligible for participation until a sufficient number of people was reached). Service users were preselected by approaching the nursing staff on the wards for a brief assessment of which service users could attend a discussion for about an hour. Informed written consent was obtained by F. S., an experienced physician, who insured that the service users were able of consenting.

With the exception of two service users, all those approached were interested in participating. All interviews and recruitment were conducted by either K. M., a female psychologist who is pursuing her dissertation on the project or the second author (F. S.), a male psychiatrist, with the other one taking notes. Both K. M. and F. S. served as research assistants and have experience in inpatient schizophrenia treatment, whereby F. S. was also experienced in conducting and analysing qualitative interviews. Training and supervision were provided by J. H., a professor of psychiatry, with many years of experience in conducting and supervising qualitative studies.

### 2.3 Data collection

Sociodemographic data were collected before the group interviews. During the interviews, a broad topic guide was used. First, the evidence of a recently published meta‐analysis on antipsychotics^12^ was presented, before wishes and expectations for a DA using such data were asked. Questions were for example: How could you imagine this data being best prepared? What content would be particularly important to you? What other aspects can you think of? Audio recordings were collected and transcribed verbatim by using a simple transcription guide.^22^

### 2.4 Data analysis

Data analysis was carried out according to the principles of thematic analysis by Braun and Clarke,^23^ using MAXQDA20. The first step was to become familiar with the content by listening to the audio recordings and reviewing the transcripts several times. Next, thoughts on the transcript were noted and what seemed significant was highlighted before each highlighted sentence was given an initial label (code). The coding was mostly inductive, but deductive coding was also used, for example by assigning labels such as ‘handling’ or ‘content of the app’. To establish a first, preliminary framework for coding, a patient focus group and a psychiatrist focus group were coded completely (40% of the data). Then the multidisciplinary research team discussed this framework and agreed on a set of codes such as ‘digital format’, ‘paper‐based decision aid’, as well as added new ones. This coding scheme was then applied to the remaining transcripts. As individual codes were again added, merged or removed, the team finally discussed once more to agree on the codes. In the next section, ‘themes’ were identified summarising various codes. For this, an analogue mind map was used to visualise codes that belong together. In two further sessions, the themes were discussed in terms of their coherence and sufficient differentiation from each other. Finally, the themes were again discussed until a consensus was reached.

## 3 RESULTS

*N* = 8 service users undergoing inpatient treatment and *n* = 10 psychiatrists attended the five focus groups. The psychiatrists were on average younger than the service users (mean age psychiatrists: 34.2 years, range 28–48; mean age service users: 39.25, range 26–63), and their gender ratio was balanced. Amongst service users, the proportion of males predominated 5 out of 8, matching the current estimated gender distribution, which indicates a higher risk of schizophrenia in males.^24^ The inclusion criterion for service users was a psychiatric diagnosis requiring treatment with antipsychotics. In addition to paranoid schizophrenia (six out of eight), also other patients (one with bipolar disorder and one with a schizoaffective disorder) were included in the study. The psychiatrists had been practising for about 6.5 years on average. Both clinics involved were teaching hospitals offering specialist training, therefore a correspondingly large number of the staff doctors were in training (see Table 2 for the summary characteristics of psychiatrists and service users).

**Table 2:** Summary characteristics of psychiatrists and service users.

Psychiatrists *n* = 10		Service users *n* = 8	
Age (mean/range; years)	34.20 (28–48)	Age (years)	39.25 (26–63)
Gender (m/w)	5/5	Gender (m/w)	5/3
Years in practice (mean, range)	6.55 (2.5–16.0)	Diagnosis	
		F20.0	6
		Other	2

Abbreviations: m, male; w, women.

**Figure 1:**
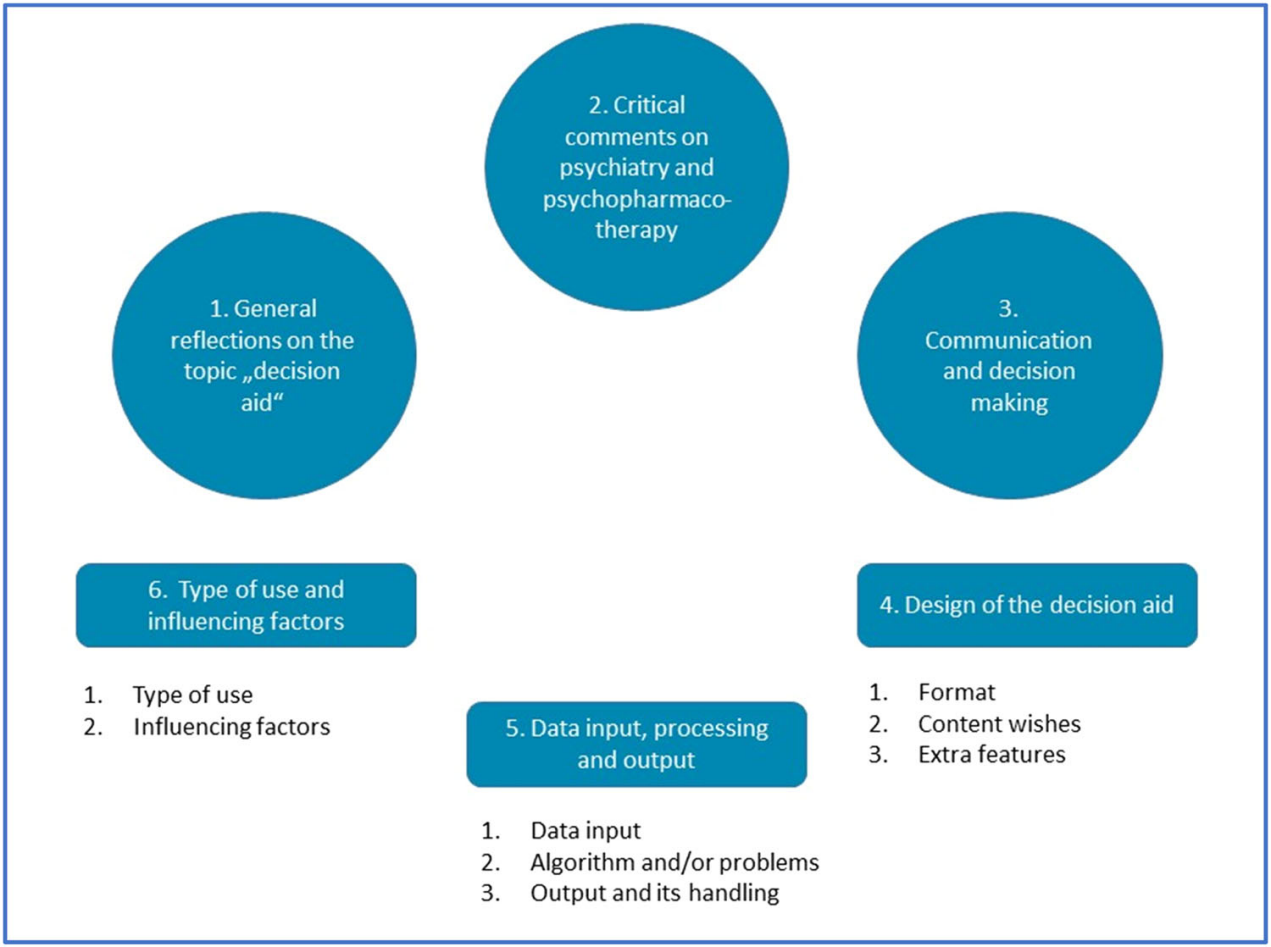
Thematic map of the identified themes.

By performing a thematic analysis with the data, six key categories were identified (see thematic map, Figure 1). In addition to wishes and expectations towards the design of the DA, the groups also shared their concerns and general opinions on the topic. Theme 1, therefore, comprises general considerations on the importance and usefulness of a DA for antipsychotics, Theme 2 displays critical comments on psychiatry and psychopharmacotherapy, while (communicative) prerequisites for the use of the DA are discussed in theme 3. Theme 4 captures specific ideas regarding the design (form and content) of the DA and theme 5 is about data input, data processing and output. Finally, suggestions were made for the application of the DA in clinical consultations and possible obstacles to its use (theme 6). Participant quotes include psychiatrist or service user group affiliation, group appointment, and gender. All themes are presented below. Codes included are reported in order of frequency, starting with the most frequently mentioned.

### 3.1 Theme 1: General reflections on the subject ‘decision aid’ and its possible use

Many participants expressed their general thoughts on the topic of DAs and their possible use, in many cases because they were confronted with the topic for the first time.

Psychiatrists and service users mentioned the desire for a reliable, easy‐to‐understand source of information on antipsychotics so that they would no longer have to resort to the summary of product characteristics or internet search engines.

Psychr: Well, what might also be nice is that sometimes we have suggested a medication and then they [service users] think a bit and they look at the package leaflet and google it and then they say ‘yes, no way, because there is such a list and it is all totally overwhelming and unorganised and there are the worst things on it’. It would be great to have an honest, realistic profile, so to speak. (G1Psychr03, male, paragraph 190)

At the same time, psychiatrists expressed their thoughts on the extent to which a DA could offer a benefit compared to overview tables from the specialist literature or searches on the internet. Hope was expressed by both sides that a DA could facilitate everyday work. Amongst others, the idea was mentioned to what extent a DA could outsource information from the doctor's consultation to be able to use the little time available effectively. Several psychiatrists expressed their uncertainty about what would actually constitute a DA. Related to this, it was unclear whether they had already used DAs previously. In two of the three psychiatrists' focus groups, the idea was also expressed that the DA could broaden the professional horizon, for example by looking at other, less frequently used drugs (e.g., G1Psychr03, male, paragraph 121).

### 3.2 Theme 2: Critical comments on psychiatry and psychopharmacotherapy

Without being explicitly asked about it, many service users reported negative experiences with psychiatric treatment in the past. Service users raised various critical aspects and negative experiences with the administration of antipsychotics, the disease and psychiatry in general. Most often they pointed out the dangers of side effects of taking antipsychotics, sometimes using the example of their own experiences. These negative consequences of drug treatment included for example tardive dyskinesia and other adverse effects. Also, some psychiatrists expressed concern about late side effects of individual drugs, for example, in the form of severe weight gain.

SU: It can also cause muscle twitching. I only had a few myself, but thank God, it went away. There are also tardive dyskinesias, which risk neuroleptics (G1SU04, male, paragraph 40)

Psychr: And I think it is very, very important that I know that if I have a side effect and it occurs and is still disturbing, how do I get out of this situation? (G1Psychr01, male, paragraph 97)

One service user expressed the wish to be offered treatment alternatives other than the administration of antipsychotics. He proposed a drug‐free treatment or suggested that patients with schizophrenia, who often also had traumatic experiences behind them, be given good, compensatory experiences. At the same time, two service users explicitly expressed their support for taking medication, amongst others emphasizing the importance of a good consultation. Several service users reported experiences with previous decision‐making processes being characterized by little involvement of the patient leading to massive sedation, or little transparency in the decision to use a particular drug. Service users discussed suffering caused by the disease itself, distrust of the prescription of high doses of antipsychotics, who is behind the administration of antipsychotics, stigmatization due to the diagnosis of schizophrenia and negative experience with reducing the drug dose without consultation of a psychiatrist. Another shared his impression of psychiatrists not liking ‘informed service users’.

### 3.3 Theme 3: Communication and decision‐making

Ideas were also expressed on communication and accountability for decision‐making, which are summarised in theme 3. Most frequently, psychiatrists named the importance of communicating with service users and using the DA only as a basis of the joint discussion.

Psychr: I mean, that should only be a basis. You have to convey somehow that that's not all, that he clicks, but that we still talk, right? That this is only a basis for what you could do. (G2Psychr01, female, paragraph 171)

Psychr: That's how it is. It doesn't replace the physician's consultation. (G2Psychr02, female, paragraph 172)

Both parties often pointed out the importance of joint decision‐making, although some service users explicitly expressed the wish to leave the final decision for or against their medication to the psychiatrist. On several occasions, service users also expressed the wish to receive information on the various medications from their practitioner. Both sides also mentioned the importance of a good physician‐patient relationship as a prerequisite for joint decision‐making.

### 3.4 Theme 4: Design of the DA

With regard to the design of the DA, the following categories became apparent: wishes for the format and content as well as desired functions that go beyond the original definition of the DA to support the choice of an antipsychotic. We called the latter category ‘extra features’. Table 3 shows the most frequently mentioned wishes for the design, broken down into the five most frequent wishes for the format, the content and ‘extra features’.

**Table 3:** Wishes regarding the format, content or related to “extra features”.

5 most frequent mentioned wishes for the format, content and “extra features” presented with the most frequent mentions first		
Format		
Digital format (“App”)	Explicitly referring to an “App” as a format for the DA or advocating a digital format *SU: Yes, I meant that you can also simply include the doctor's report in the app in order to adjust the app to me as a person. With age, name, weight and the doctor's report in general, what cases you have had, etc. So that taking the pills is easier. So that you know what could happen, so that the app can roughly estimate, calculate what will come out*. (G1SU1, male, paragraph 69)	SU & Psychr
Various display options	More than one version or interface of the DA (e.g. the idea of extension, reduction options, different logins for service users and psychiatrists, etc.) *Psychr: I think there can simply be two user‐modes. One for service users to log‐in and one for doctors to log‐in, so you can start with the simple things for the drugs and then click on them and then get more detailed information and maybe also a quick overview and then again in detail when you can deal with it more closely and have more time*. (G2Psychr02, female, paragraph 83)	SU & Psychr
Simple presentation, clarity	The desire for clarity in presentation *Psychr: Not all physicians are familiar with these study images but it has to be more instinctive for the service users, so with colours, with bars, because not to forget that the service users, even if they are still being cared for by us, are not yet in full remission. Or are just in remission and you really just have to work with more colours, with more shapes, perhaps, so that they can also perceive it better visually*. (G2Psychr02, female, paragraph 33)	SU & Psychr
Analogue, on paper	The explicit wish for an analogue version of the DA *SU: Well, yes, more on paper, you don't always have access to a website or whatever*. (G2SU03, male, paragraph 134)	SU & Psychr
Avoid deficit orientation	The concern that mentioning too many side‐effects or focusing on the negative aspects of a drug could lead to a loss of motivation and/or the recommendation to emphasise positive aspects of drug treatment. *Psychr: One thing that would still be important to me is when I show this to the patient or when I then have the possibility to print out their profile, then it shouldn't be too deficit‐oriented because then it's probably like this: I have the effect size on positive symptoms, on negative symptoms and then there are all the side effects‐ and that probably overwhelms in proportion and then the patient will just say like with the package insert where everything is red: “yes sorry people, I'm not taking that drug”. So there are two things that are green and the rest is simply a disaster and no matter which drug they would say “it's all a disaster, I don't want to take any of them now”*. (G1Psychr01, male, paragraph 124)	Psychr
Content		
Information on antipsychotics	Wish that the DA offers a (short) summary of each drug. *Psychr: Yes, and another function –I'm thinking of people who are dealing with this for the first time‐ could be to say that you can read everything again as a profile in the continuous text about the individual neuroleptics. So, not as, I don't know, chapters, pages long, but also concise, somehow. So that you can still access it somehow, still have a short text on the neuroleptics*. (G3Psychr02, female, paragraph 140)	SU & Psychr
Reversibility of side effects	The desire for guidance on the management of potential side effects in the DA. *Psychr: Reversibility. That when I would say, “okay that side effect, I'll risk it, that I know that I will gain weight but if I then know that 95% of the weight will never come off, then I might deal with it differently”. Or in the case of sexual dysfunction, that I know that it is potentially reversible. How many of them manage to get rid of it after stopping the medication? That would also be extremely important for me. I always assess the risk consciously. On the one hand I have to decide on a medication, but then I also have to say that the side effects show up, even if it has perhaps not felt that important to the patient‐ I take the risk and then I also have to know, if I should change the medication, how sure I can be that these side effects are then also limited in time. I would find that important*. (G1Psychr01, male, paragraph 28)	Psychr
Side‐effect profile	The desire for a risk profile (“side‐effect profile”) of each antipsychotic *SU: Or you can, for example, if we just say weight gain, you can of course also say: “okay, there is weight gain and these and those drugs do that” and then compare the options. So which ones have a very high probability and which ones, as we have just discussed, have a lower probability?* (G1SU04, female, paragraph 91)	SU & Psychr
Clinical relevance of side‐effects	Wish for information on the clinical relevance of side‐effects. *Psychr: And when it comes to weight‐ sorry to add‐ that is more understandable to patients than the QTc‐ whether it [note: the weight gain] is 3 kilograms, 5 kilograms or 10 kilograms*. (G1Psychr02, male, paragraph 27)	Psychr
Information on side‐effects	Wish for detailed information on the individual side‐effects. *SU: So an extra (.) umbrella term or a column where you can then click on it and you can see how the drug works. There is like a table: “there is the mode of action, there are side effects, there is just something else or whatever and then you can click in each case an then it is displayed*. (G1SU03, female, paragraph 109)	SU & Psychr
Extra features		
Interaction checks and contraindications	Desire for flagging of interactions and/or warnings of contraindications. *Psychr: But I would think that it would be good to see it somehow similar to PSIAC [note: an interaction software frequently used in Germany], is it now an absolute contraindication, if I now (.) ‐the extreme example‐ have someone with cardiomyopathy and then I want to give them clozapine, so that won't happen*. (G1Psychr05, female, paragraph 169)	SU & Psychr
Monitoring options	Wish to include reminders for control treatments such as EEG checks, blood count checks, etc in the DA *Psychr: That everything is included‐ so with the weight, that you can monitor and enter it if you want, or lab values, maybe cholesterol values or blood sugar values that you can enter that, or save it, or whatever*. (G1Psychr04, female, paragraph 117)	Psychr
Link to other databases	Other tools that are already established in clinical practice and currently play a role in medication decision‐making (e.g. “AID Clinic”, PSIAC) and/or the desire to link the DA with their contents. *Psychr: Well, there is, for example, “psychiatry‐to‐go” or these classic ones, where all the side effects are shown in a clock and then the points go up in different heights and then you have such a graphic construct, and so that's something, for example, I always have it printed out and lying there and then, before I prescribe something, I look at it again, and I haven't really forgotten anything. So that is, I think, already very simply represented graphically*. (G1Psychr01, male, paragraph 57)	Psychr
Combination therapy	Combination with other drugs to be taken into account in the DA *Psychr: So if we are in the request phase, then I would also like to see a tool for combination therapies*. (G3Psychr03, male, paragraph 125)	Psychr
Dose ranges	Need to know the (maximum) possible dose of the antipsychotic in question. *I: […] And you (turns to P2) mentioned something else important. The dosage, that that would also be important to you?* *SU: The dosage, that if (.) at the beginning, for example, two different pills are combined, maybe. Every person is different and (.) you can try not to use such strong doses, yes?* (G2SU02, female, paragraph 72)	SU

*Note*: SU = statements from service users (blue), Psychr = based on statements from psychiatrists (white), SU & Psychr = statements of this category were made by both psychiatrists and service users (grey).

Abbreviations: DA, decision aid; EEG, electroencephalogram.

### 3.5 Theme 5: Data input, processing and output

**Figure 2:**
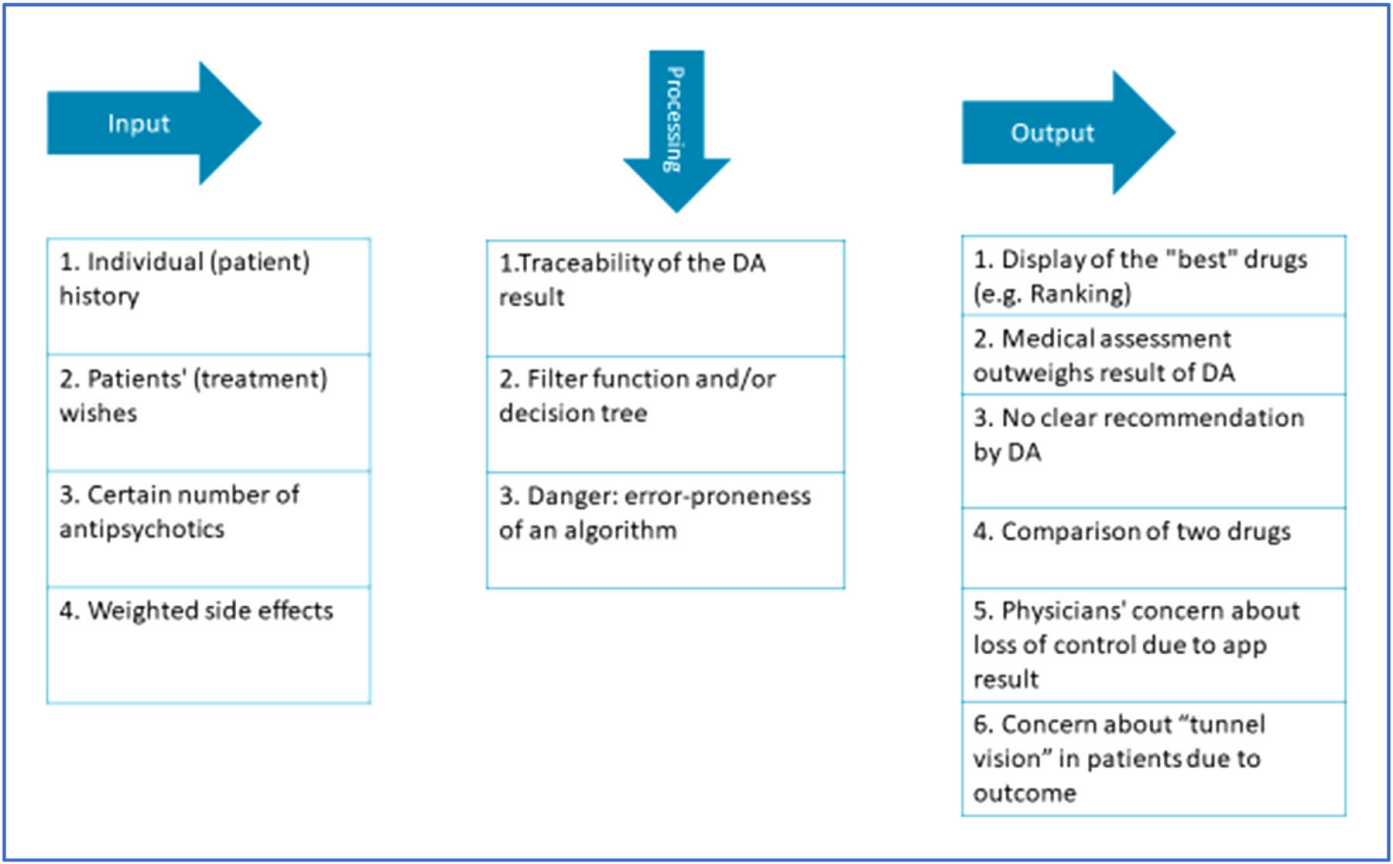
Thoughts on possible input and output of the decision aid (DA).

Thoughts were expressed on what information should be included as input, how this information could be processed and how a possible output could be presented, including possible difficulties associated with it. Figure 2 illustrates the ideas on input and output sorted by frequency of utterance. Most often, the individual patient history was mentioned as a desired input of the app:

Psychr: You could personalise it a bit more. So, depending on the patient's pre‐existing conditions … then if they have diabetes, some antipsychotics are excluded, if they are older, some are excluded, was there a change in medication in the record? Then that as well. (G3Psychr01, male, paragraph 50)

#### 3.5.1 Algorithm and/or issues in processing

With regard to the DA's data processing, psychiatrists suggested a decision tree or a filtering mechanism. The suggestion would lead to ‘deselecting’ antipsychotics that are unsuitable according to the service user profile. Above all, the transparency of the algorithm was repeatedly emphasized.

Psychr: Such a [decision] tree, so that the patient can retrace how we came to the decision. […] Also the steps to get there could be explained. That you can also go back again if you come out where you don't want to come out, or you can have another look at what you can perhaps accept as a side effect. (G3Psychr2, female, paragraph 46‐48)

Psychiatrists were critical of any algorithm that is supposed to lead to a clear treatment recommendation in such complex decisions.

#### 3.5.2 Output and handling

Most frequently mentioned in connection with the output of the DA was the wish for the recommendation of ‘a best’ or ‘several best’ antipsychotics.

Psychr: Maybe you could go through that by yourself in an app and choose. I think it's good to have some knockout criteria where you can say this side effect is not acceptable or that is omitted because of renal insufficiency and so on. You can then go through that with the patients themselves, like a tree. You just start and end up with a drug. Maybe with an alternative. (G3Psychr02, female, paragraph 43)

SU: I also think that a specific proposal would be useful and that you then go to the doctor with it. (G1SU03, male, paragraph 218)

Much emphasis was placed by both parties to leave the final decision on medication to the psychiatrist rather than to the DA. Various fears were expressed by psychiatrists in the context of a clear drug recommendation, such as the concern that service users might become fixated on the outcome and that they as physicians might be deprived of control over the decision‐making process; although the objection was also raised several times that this need not be a concern in the context of shared use. Occasionally, psychiatrists also suggested not making a recommendation but presenting the different antipsychotics on an equal footing.

### 3.6 Theme 6: Type of use of the DA and influencing factors

Theme 6 includes perceptions from both sides about who should primarily use the DA. In this context, psychiatrists also expressed various concerns or possible barriers to use.

#### 3.6.1 Type of use

The idea most frequently mentioned by service users and physicians was to hand out the DA to service users for their sole use before or after a joint discussion with the psychiatrist.

SU: In the beginning with the doctor, certainly.

I: Um. Why? Why wouldn't you like to do it on your own?

SU: Yes, then definitely also by myself. First with the doctor, to see their assessment in the consultation, and then definitely alone. So, I would say, first a conversation with the doctor and then access it alone and also work with it. (G1SU04, female, paragraph 192‐194)

However, both implicitly and explicitly, there was also an expectation on both sides to provide the DA only to the psychiatrist (who submits the results to the SU). However, the desire for joint use of the DA was also frequently found on the part of both sides: a single suggestion on the service user side also brought forth the idea of having the doctor‐patient communication take place purely digitally.

#### 3.6.2 Influencing factors

Only psychiatrists mentioned possible hindering factors for the use of the DA by service users. These included, for example, difficulties in dealing with the DA or possibly limited openness due to psychopathological characteristics of the disease such as cognitive deficits or mistrust towards staff or media. Personality traits (openness) and other characteristics such as ‘age’ were also brought into play as influencing factors for use. Reflections on the timing of the use of the DA were mentioned, which were associated with very different demands on the design from a psychiatrist's point of view. In particular, the use of a DA in the acute stage of the disease was questioned.

Psychr: I think that actually only a certain patient clientele ‐ at a certain point of the illness ‐ actually benefits from it. For instance, I don't think you could say across the board that everyone who needs neuroleptics does. I also believe that perhaps ‐ I don't know ‐ 50% to 60% of the doctors might benefit from it and that you then also have to look at which patients can also process it in this way. So, who has the intellect, the age, whatever, also is able to deal with apps and so on ‐ many have a mistrust and are psychotic – of such technical things where you have to enter some data in the screen. So, I think that in a very practical way, it is often, I don't know, connected with inhibition. (G3Psychr02, female, paragraph 114)

## 4 DISCUSSION

### 4.1 Findings and current state of knowledge

Our findings suggest that there are many hopes but also concerns about the use of DAs in the (acute) inpatient treatment of schizophrenia, with psychiatrists and service users setting different priorities (see Table 4). What united both sides was the desire for an easy‐to‐understand source of reliable information, which seems to indicate a general openness to the use of an evidence‐based DA. In particular, the ability to customize the complexity by fading in individual content or setting up different logins for different users seemed to be a decisive advantage of a digital tool. However, the desire for two formats (digital and analogue) was also expressed, to provide easier access for older service users or those with acute illnesses. In addition to information on the drugs, information on side effects was most frequently requested, including a side effect profile, the clinical relevance of side effects and information on their reversibility. Consistent with the findings of Kaar et al.,^25^ service users mentioned negative experiences with medications more often than positive ones and did so more frequently than psychiatrists. The most frequently mentioned idea about the output of the app—especially from psychiatrists—was that it should give a clear recommendation for a specific, most suitable medication after use. In this context, however, the danger was also pointed out that physicians may then only be able to influence the treatment decision to a limited extent if the users develop ‘tunnel vision’ as a consequence of the app's results. Psychiatrists, therefore, often also called for a gradual phasing out of medication that would lead to a comprehensible end result.

**Table 4:** Differences and common grounds in wishes and expectations towards the decision aid (DA) of psychiatrists and service users.

Patients	Both	Psychiatrists
**Importance of treatment alternatives** **The doctor explains to the patient** **Dose ranges** **Patients communicate online with physicians** *Advocacy for antipsychotics* *Negative experience with decision‐making processes* *Mistrust of psychiatry* ‘*A shitty disease*’ *Stigmatisation* *Negative experience with own re‐dosing* *Negative reaction to informed patients* ‘*The doctor knows best*’	**The desire for a reliable source of information** **DA should make everyday work easier** **Dangers of antipsychotics** **Shared decision desired** **Digital format (**‘**App**’**)** **Various display options** **Simple presentation/clarity** **Analogue, on paper** **Information on antipsychotics** **Side‐effect profile** **Information on side‐effects** **Interaction checks and contraindications** **Individual patient history** **Patient's treatment wishes** **Certain number of antipsychotics** **Sole use of the DA through patient** **Physician uses the DA** **Joint use of the DA** *Importance of a good doctor‐patient relationship* *Added value of a DA*?	**Importance of medical communication in decision‐making** **Avoid deficit orientation** **Reversibility of side‐effects** **Clinical relevance of side‐effects** **Monitoring options** **Link to other databases** **Combination therapy** **Weighted side effects** **Traceability of the DA result** **Filter function and/or decision tree** **No clear recommendation by DA** **Comparison of two drugs** **Other patient characteristics** *Physicians concern about loss of control due to app result* *Concern about* ‘*tunnel vision*’ *due to outcome* *Psychopathology and handling of the DA* *Considerations on the time of use* *Uncertainty about what a DA is* *Previous experience with DAs* *DAs broaden the professional horizon* *Danger: error‐proneness of an algorithm*

*Note*: Wishes/expectations for the DA are indicated by the bold font, and concerns or other thoughts on the topic are in italics.

Both sides expressed the idea that the app could be used by service users alone before or after the consultation, for example, to save time, but both sides expressed the desire not to refrain from a joint consultation. Possible impeding factors for the use of DA were almost exclusively mentioned by psychiatrists who, on the one hand, consider it very important to communicate with service users as early as possible, but on the other hand, are also sceptical about the use of evidence‐based DA in the early stages of an acute psychotic episode. Possibly, such statements indicate a certain stigmatizing tendency on the part of psychiatrists, which has also recently been suspected as a possible obstacle to SDM, especially in contact with service users with SMI.^26^ At the same time, service users frequently expressed a desire for the presentation to be as simple and clear as possible. This highlights the complexity of the situation and the need to convey as much information as possible without being overwhelming. Some service users also expressed the wish to leave the final word to the physician. This could be an expression of self‐stigma, which also seems to play a role in people with mental illness.^27^ On the other hand, this could also be related to another important issue raised by Zisman‐Ilani et al.,^9^ who propose a new conceptualization of SDM in the treatment of individuals with SMI: the so‐called ‘shared risk’ approach. Indeed, it should not be forgotten that treatment decisions in acute psychiatric settings often involve risk‐taking on the part of both the patient and the clinician, for example, when symptom exacerbation may be accompanied by suicidality or homicidality, which could entail liability consequences and thus be a potential impeding factor for the implementation. They, therefore, advocate for distinguishing between decisions of higher risk and those of lower risk as well as communicating this clearly and adapting SDM for people with SMI accordingly.

### 4.2 Implications for clinical practice and the development of DAs

Based on our interviews, we draw the following conclusions regarding the development of a DA for antipsychotics in the inpatient setting: A digital tool seems to be the most suitable to combine the different expectations of both sides.

We propose an app that can be used and viewed separately by both physicians and service users, but at the same time has a common, scalable interface.

Our proposal is that patients receive well‐prepared information that can be obtained without the presence of psychiatrists and that provides basal information about treatment options and the disease. Experience knowledge with different drugs should also be queried. In parallel, physicians should be able to provide information on possible interactions or comorbid diseases. Both the medical information and the empirical knowledge are to be recorded in the app and can be taken into the joint discussion. There, the evidence should be easily processed and colourfully presented to stimulate joint discussions. Different information should be viewed depending on psychopathology, cognitive abilities, and information needs, by selecting different tabs.

In particular, the scalability of information seems to us to be most easily implemented in the form of a digital tool, as well as best suited to meet the specific requirements in the acute setting.

### 4.3 Limitations

Several limitations of this study need to be addressed. Contrary to what has recently been emphasized as ‘triadic shared decision‐making’,^28^ we limited the sample to service users and psychiatrists as the main stakeholders of the decision against or for antipsychotics, although caregivers and family members also play an important role in treatment planning in the inpatient setting.^29^ A certain selection bias must be noted in the recruitment of patients. By asking the nurses about their assessment of the patient's ability to concentrate and selecting suitable candidates based on this, those with stronger positive symptoms such as inner restlessness or concentration problems were excluded. As the research group was located at one of the recruitment centres where half of the interviews took place, biases in the response behaviour of patients and medical colleagues can also not be ruled out. In addition, we showed subjects study results that should find a place in future DA but did not present an actual, already existing tool. Another restriction is that the coding of the data material was carried out by one investigator alone. However, regular interdisciplinary team meetings were held to ensure intersubjectivity in the key steps of the data analysis, such as setting up the coding scheme and eliminating ambiguities.

## 5 CONCLUSION

Although patient DAs are becoming increasingly popular in the field of schizophrenia treatment, there is still the need to develop and test new tools that address the complexity of the decision on antipsychotic medication and eliminate hindering factors responsible for the poor implementation of SDM in psychiatry, especially for people with SMI. For this reason, time constraints as well as cognitive deficits, amongst others, should be considered. Nevertheless, the aim must be to provide those involved in the decision‐making process with the highest possible data quality to enable a personalised, well‐informed decision. Consulting the main stakeholders involved in this decisional process, we gained important insights to develop an evidence‐based DA with the potential to be highly useful to both parties, and also feasible in future implementation.

## AUTHOR CONTRIBUTIONS

Katharina Müller planned the study, managed data acquisition, interviewed participants, planned the analysis, analysed the results and drafted the manuscript. Florian Schuster planned the study, managed data acquisition, interviewed participants, analysed the results and critically revised the manuscript. Prof. Silvia Krumm planned and supervised the project together with Prof. Johannes Hamann with her expertise as a qualitative researcher critically revised the manuscript. Prof. Stefan Leucht planned the study, managed data acquisition and critically revised the manuscript. Spyridon Siafis planned the study and critically revised the manuscript. Stephan Heres planned the study, managed data acquisition and critically revised the manuscript. Peter Brieger planned the study, managed data acquisition and critically revised the manuscript. Johannes Hamann planned the study, managed data acquisition, supervised the whole process of data acquisition and analysis as a qualitative expert, analysed the results and critically revised the manuscript.

## CONFLICTS OF INTEREST STATEMENT

In the last three years Stefan Leucht has received honoraria as a consultant and/or advisor and/or for lectures and/or for educational material from Alkermes, Angelini, Eisai, Gedeon Richter, Janssen, Lundbeck, Medichem, Medscape, Merck Sharpp and Dome, Mitshubishi, Neurotorium, NovoNordisk, Otsuka, Recordati, Roche, Rovi, Sanofi Aventis, TEVA. Johannes Hamann received lecture honoraria from Janssen, Otsuka, Lundbeck and Rovi In the past three years, Stephan Heres has received lecture honoraria from Johnson & Johnson and Otsuka/Lundbeck, as well as honoraria for scientific advisory boards in clinical trials for TEVA, ROVI and KYE.

## Supporting information

Supplementary information.

## Data Availability

The data that support the findings of this study are available from the corresponding author upon reasonable request.
