# Supplementary material for: Informing the development of a decision aid: Expectations and wishes from service users and psychiatrists towards a decision aid for antipsychotics in the inpatient setting
Source: Health Expect. 2023 Mar 14;26(3):1327–38. doi: 10.1111/hex.13749 (PMC10154827; doi:10.1111/hex.13749)
Supplement: Supplementary file 1 — Supplementary information. [file HEX-26--s001.docx]

**Informing the development of a decision aid: Expectations and wishes from service users and psychiatrists towards a decision aid for antipsychotics in the inpatient setting**

Katharina Müller^1,2^, Florian Schuster^1,3^, Silvia Krumm^4^, Stefan Leucht^1^, Spyridon Siafis^1^, Stephan Heres^2^, Peter Brieger^2^ and Johannes Hamann^1,5^

Table of Contents

[1.Reporting Checklist 3](#_Toc110801248)

[2.Topic Guide 6](#_Toc110801249)

[3.Template of informed consent 10](#_Toc110801250)

[4. References 16](#_Toc110801251)

# **1.Reporting Checklist**

*COREQ Checklist*

The COREQ checklist was used for the reporting of the methods section (Tong et al., 2007):

| No | Item | Guide questions/description |
| --- | --- | --- |
| **Domain 1: Research team and reflexivity**  *Personal Characteristics* | | |
| 1. | Interviewer/facilitator | Which author/s conducted the interview or focus group? |
| 2. | Credentials | What were the researcher’s credentials? E.g. PhD, MD |
| 3. | Occupation | What was their occupation at the time of the study? |
| 4. | Gender | Was the researcher male or female? |
| 5. | Experience and training | What experience or training did the researcher have? |
| *Relationship with participants* | | |
| 6. | Relationship established | Was a relationship established prior to study commencement? |
| 7. | Participant knowledge of the interviewer | What did the participants know about the researcher? e.g. personal goals, reasons for doing the research |
| 8. | Interviewer characteristics | What characteristics were reported about the interviewer/facilitator? e.g. Bias, assumptions, reasons and interests in the research topic |
| **Domain 2: study design**  *Theoretical framework* | | |
| 9. | Methodological orientation and Theory | What methodological orientation was stated to underpin the study? e.g. grounded theory, discourse analysis, ethnography, phenomenology, content analysis |
| *Participant selection* | | |
| 10. | Sampling | How were participants selected? e.g. purposive, convenience, consecutive, snowball |
| 11. | Method of approach | How were participants approached? e.g. face-to-face, telephone, mail, email |
| 12. | Sample size | How many participants were in the study? |
| 13. | Non-participation | How many people refused to participate or dropped out? Reasons? |
| *Setting* | | |
| 14. | Setting of data collection | Where was the data collected? e.g. home, clinic, workplace |
| 15. | Presence of non-participants | Was anyone else present besides the participants and researchers? |
| 16. | Description of sample | What are the important characteristics of the sample? e.g. demographic data, date |
| *Data collection* | | |
| 17. | Interview guide | Were questions, prompts, guides provided by the authors? Was it pilot tested? |
| 18. | Repeat interviews | Were repeat interviews carried out? If yes, how many? |
| 19. | Audio/visual recording | Did the research use audio or visual recording to collect the data? |
| 20. | Field notes | Were field notes made during and/or after the interview or focus group? |
| 21. | Duration | What was the duration of the interviews or focus group? |
| 22. | Data saturation | Was data saturation discussed? |
| 23. | Transcripts returned | Were transcripts returned to participants for comment and/or correction? |
| **Domain 3: analysis and findings**  *Data analysis* | | |
| 24. | Number of data coders | How many data coders coded the data? |
| 25. | Description of the coding tree | Did authors provide a description of the coding tree? |
| 26. | Derivation of themes | Were themes identified in advance or derived from the data? |
| 27. | Software | What software, if applicable, was used to manage the data? |
| 28. | Participant checking | Did participants provide feedback on the findings? |
| *Reporting* | | |
| 29. | Quotations presented | Were participant quotations presented to illustrate the themes / findings? Was each quotation identified? e.g. participant number |
| 30. | Data and findings consistent | Was there consistency between the data presented and the findings? |
| 31. | Clarity of major themes | Were major themes clearly presented in the findings? |
| 32. | Clarity of minor themes | Is there a description of diverse cases or discussion of minor themes? |

# **2.Topic Guide**


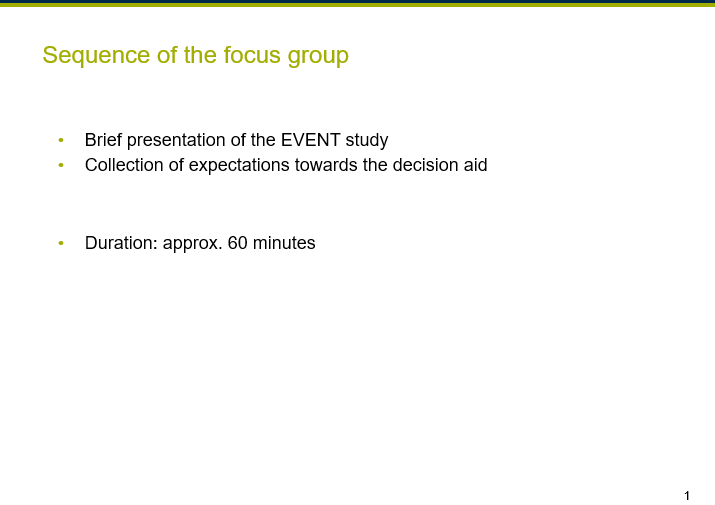


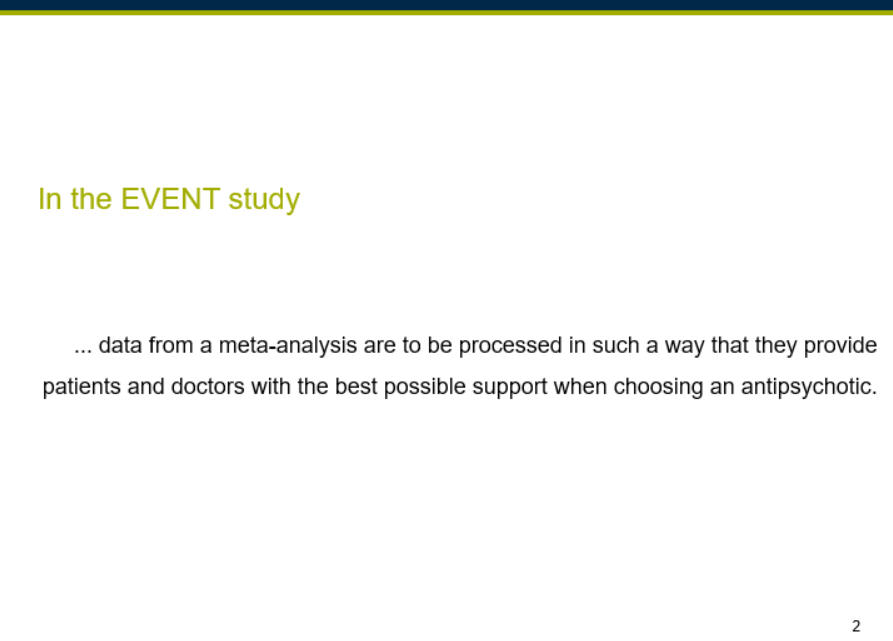


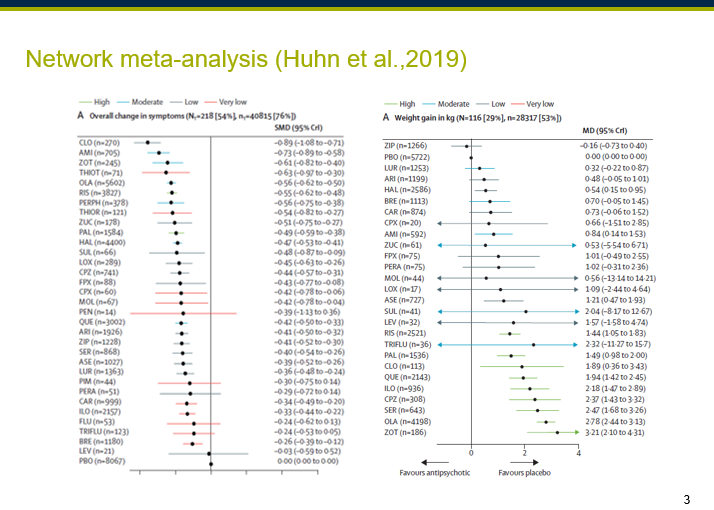


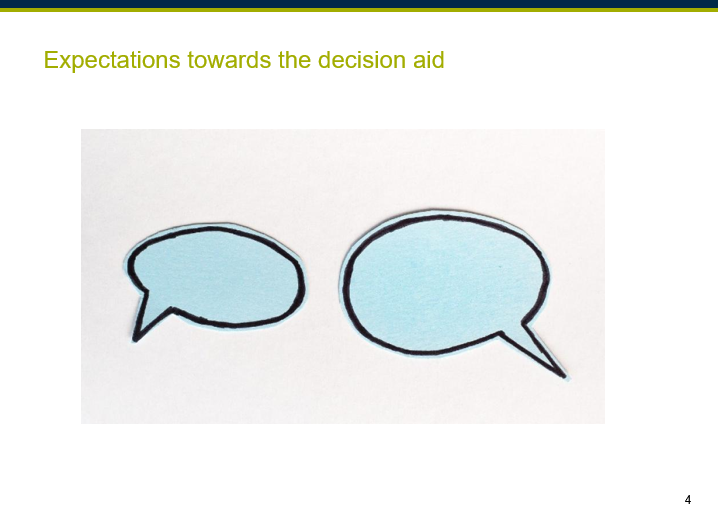


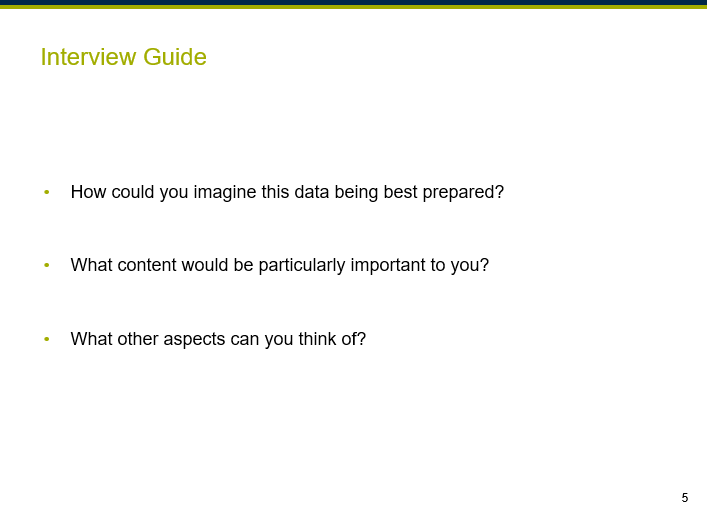


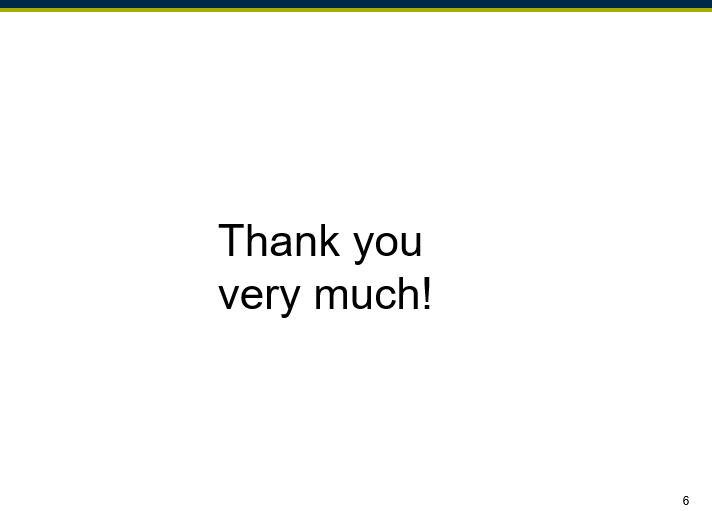


# **3.Template of informed consent**

# **4. References**

Tong, A., Sainsbury, P., & Craig, J. (2007). Consolidated criteria for reporting qualitative research (COREQ): A 32-item checklist for interviews and focus groups. *International Journal for Quality in Health Care*, *19*(6), 349–357. https://doi.org/10.1093/intqhc/mzm042
